# Patient-centered outcomes with subcutaneous immunoglobulin use for infection control in primary and secondary immunodeficiencies: data of a GEIE Spanish Registry

**DOI:** 10.3389/fimmu.2025.1532367

**Published:** 2025-02-14

**Authors:** Sandra Martínez Mercader, Victor Garcia-Bustos, Pedro Moral Moral, Carmen Martínez Buenaventura, Elisa Escudero Vergara, María Carmen Montaner Bosch, Héctor Balastegui-Martín, Sonia Galindo Maycas, Berta Palací Mur, Marian Escobar Palazón, María Moreno Mulet, Ignacio Campanero Carrasco, Alicia López, Carlos Daniel Hernández Ruiz, Laura Ruiz-López, Rocío Guzmán Guzmán, Marta Dafne Cabañero-Navalon

**Affiliations:** ^1^ Primary Immunodeficiencies Unit, Department of Internal Medicine, University and Polytechnic Hospital La Fe, Valencia, Spain; ^2^ Severe Infection Research Group, Health Research Institute La Fe, Valencia, Spain; ^3^ Research Group of Chronic Diseases and HIV Infection, Health Research Institute La Fe, Valencia, Spain; ^4^ Pediatric Infectious Diseases and Immunodeficiencies Unit, Vall d’Hebron University Hospital, Barcelona, Spain; ^5^ Area of Immunology - Multidisciplinary Day Hospital, Gregorio Marañón General University Hospital, Madrid, Spain; ^6^ Immunology Day Hospital Unit, Reina Sofía University Hospital, Córdoba, Spain; ^7^ Onco-Hematology Day Hospital, La Paz University Hospital, Madrid, Spain; ^8^ Internal Medicine Day Hospital, Doctor Negrín University Hospital of Gran Canaria, Gran, Canaria, Spain; ^9^ Clinical Immunology and Primary Immunodeficiencies Unit, Pediatric Allergy and Clinical Immunology Department, Sant Joan de Déu Hospital, Barcelona, Spain; ^10^ Day Hospital, Son Espases University Hospital, Palma de Mallorca, Spain

**Keywords:** subcutaneous immunoglobulin (SCIg), primary immunodeficiency (PID), secondary immunodeficiency (SID), patient-centered outcomes, quality of life

## Abstract

**Background and aim:**

Subcutaneous immunoglobulin (SCIg) has emerged as an alternative to intravenous administration for patients with primary (PID) and secondary immunodeficiencies (SID), offering benefits such as fewer systemic adverse reactions and greater patient autonomy. However, comprehensive real-world data on SCIg use, including clinical and patient-centered outcomes, remain scarce. This study, conducted by expert immunodeficiency nursing teams, assesses the clinical characteristics, reported adverse effects, and quality-of-life outcomes associated with SCIg therapy with different formulations in patients with PID and SID across Spain.

**Methods:**

A multicenter, cross-sectional study was conducted across 8 immunodeficiency nursing units in Spain, involving 223 adult patients treated with SCIg from 2004 to 2024. Data on demographics, comorbidities, SCIg treatment characteristics, reported adverse events, and quality-of-life metrics (EuroQol-5D-3L, Gijón Scale) were collected and analyzed.

**Results:**

The cohort (61.4% female, mean age: 47.1 years) included 65% PID patients, with common variable immunodeficiency being the most frequent diagnosis (39.8%). SCIg demonstrated good tolerability overall, with no significant differences in global adverse event rates between facilitated 10% (fSCIg) and 20% formulations. However, 10% fSCIg was associated with higher reported frequencies of mild local rash (58.7% vs. 36.9%, p=0.002) and fever (10.6% vs. 1.7%, p=0.01). Quality-of-life scores indicated minimal limitations in mobility and self-care, with a mean subjective health rating of 72.7/100. Patients using 20% SCIg required fewer educational sessions for self-administration compared to the 10% group.

**Conclusion:**

The different SCIg formulations in this large, multicenter cohort was effective and generally well-tolerated, supporting its use for maintaining adequate IgG levels and promoting patient independence in PID and SID. The study’s findings advocate for tailored approaches that optimize patient satisfaction and address individual needs, emphasizing the critical role of dedicated immunodeficiency nursing teams in ensuring safe, effective, and patient-centered SCIg administration.

## 1 Introduction

Immunoglobulin replacement therapy (IgRT) is an essential intervention for patients with primary (PID) and secondary immunodeficiencies (SID) who experience impaired antibody production. IgRT has demonstrated efficacy in reducing infection rates and severity, preventing organ damage, and lowering mortality (1, 2). Initially introduced in the 1950s as intramuscular preparations for humoral PID, immunoglobulin therapy evolved significantly with advancements in plasma fractionation, leading to the development of intravenous immunoglobulin (IVIg) in the 1980s. In Spain, subcutaneous immunoglobulin (SCIg) became available in 2004, with facilitated 10% SCIg (fSCIg) formulations introduced in 2017 (3, 4) (AEMPS, Hizentra; AEMPS, Hyqvia).

Although IVIg and SCIg have comparable efficacy, SCIg is associated with several advantages, including fewer systemic side effects, improved quality of life, and reduced healthcare costs (5, 6). A meta-analysis of 24 studies involving 946 PID patients found that transitioning from IVIg to SCIg resulted in higher serum IgG levels and fewer adverse events, with no increase in infection rates (7). Furthermore, home-based SCIg administration has been shown to yield economic benefits compared to hospital-based IVIg treatment (8). SCIg therapy also enhances patient autonomy and flexibility, as it can be self-administered at home following comprehensive training from expert immunodeficiency nurses. This specialized training is critical for safe and effective self-management, addressing infusion procedures, adverse event mitigation, and preventive measures.

Despite these advantages, SCIg remains underutilized in Spain and Europe, even within specialized centers (9). Inconsistencies persist across national guidelines regarding SCIg indications for SID, and discrepancies remain between recommendations and clinical practice (10, 11). Furthermore, even after three decades since SCIg’s introduction, there remains a significant lack of international real-world data on its use, particularly in SID populations, insights that are crucial for optimizing and expanding its application in this group (12). Comprehensive epidemiological and clinical insights into SCIg therapy for both PID and SID are notably limited.

This study aims to provide a comprehensive cross-sectional assessment of the use of both 20% and 10% fSCIg preparations in specialized PID nursing units across Spain, evaluating clinical and sociodemographic characteristics, administration practices, therapeutic outcomes, adverse effects, and the impact on quality of life, including social, economic, and psychological dimensions for both PID and SID patients.

## 2 Material and methods

### 2.1 Study design and ethical statement

**Figure 1:**
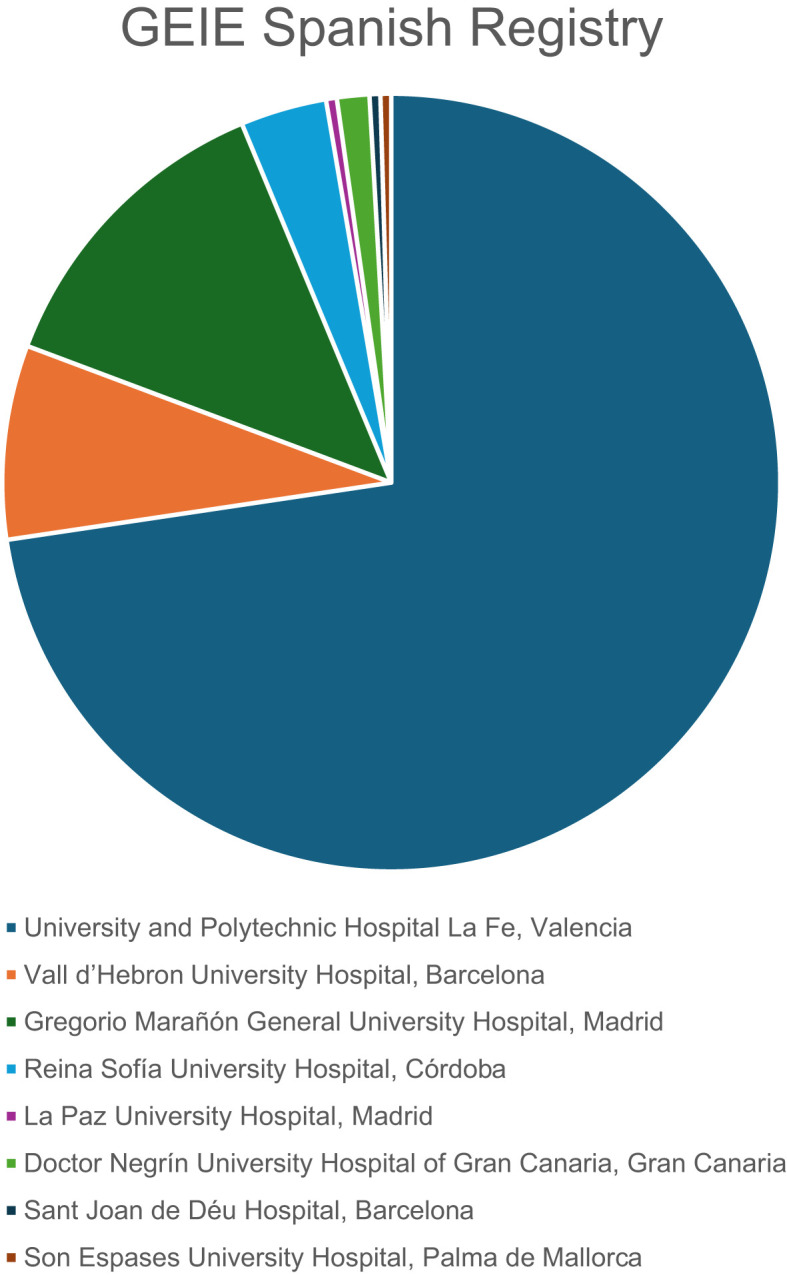
Distribution of patients contributed by each hospital in the GEIE Spanish Registry study.

A multicenter, retrospective study was conducted in Spain between November 2023 and August 2024, involving patients aged 14 years and older with PID and SID who had received or were receiving SCIg since its introduction in Spain in 2004. Patients were prescribed SCIg based on clinical severity and infection burden, as determined by their reference clinical practitioners and clinical guidelines. Eight immunodeficiency units from different hospitals participated in the development of the GEIE Spanish Registry, an initiative led by the Immunodeficiency Nursing Group of the Spanish Society of Immunology (**Figure 1**). Patients with a diagnosis of PID or SID who had received SCIg (including facilitated SCIg) for at least one month during their clinical course, were considered eligible for inclusion.

The protocol of the GEIE Spanish Registry was independently approved by all institutional Ethical Committees of each participating hospital under their corresponding registry codes. Anonymity and data confidentiality of all included patients were ensured in accordance with the Spanish regulation of observational studies. The work was performed according to the Declaration of Helsinki and followed the STROBE guidelines.

### 2.2 Data collection and variables

The GEIE Spanish Registry systematically compiles sociodemographic, epidemiological, and clinical data, including patient comorbidities, characteristics of SCIg therapy, technical aspects of SCIg administration, reported adverse effects, and a comprehensive evaluation of the patients’ social, economic, and psychological status. Data were collected retrospectively from medical records and supplemented with information from patient interviews using predefined questionnaires, especially regarding to adverse events and quality of life impacts. All data were obtained by expert nurses specialized in immunodeficiency care.

Demographic data collected included sex, current age, date of birth, age at treatment initiation, diagnosis (PID or SID), and, in the case of PID, the specific diagnosis. Comorbidities were also documented, including immune dysregulation-related conditions such as autoimmune cytopenias, organ-specific autoimmunity, and a history of benign lymphoproliferation affecting the spleen or lymph nodes. The presence of bronchiectasis or parenchymal lung disorders was recorded, as well as any history of chronic liver disease, enteropathy, or dermatological conditions. Additionally, a history of solid or hematologic malignancies, solid organ transplantation, or hematopoietic stem cell transplantation (HSCT) was registered, along with any drug allergies or other allergic conditions.

The use of oral or subcutaneous anticoagulants and antiplatelet therapies was documented. Baseline total IgG levels prior to SCIg initiation and trough IgG levels during treatment were recorded. Information was collected on whether patients were actively receiving SCIg at the time of inclusion, or if they had switched to IVIg or discontinued IgRT, including reasons for these changes. Local adverse reactions to SCIg (erythema, pain, pruritus, swelling, fluid leakage, hematoma, ulceration, necrosis), as well as systemic adverse reactions to SCIg (headache, asthenia, fever, arthralgia, myalgia, generalized rash, wear-off effect), were recorded according to cumulative patient experience.

Details of SCIg administration were captured, including the type of SCIg product, the site of administration (outpatient clinic, external consultation, or home), dosage in grams per week, frequency of administration, infusion volume (for both 20% and 10% concentrations), infusion time, number of infusion sites, and needle gauge and length. The patient’s abdominal circumference and the number of training sessions required for self-administration were also documented.

Socioeconomic data collected included the distance from the patient’s home to the hospital and their employment status. The Gijón Scale (13) was applied to assess family situation, including living arrangements, monthly income, housing conditions, social relationships, and social network support. Additionally, patients completed the EuroQol-5D-3L questionnaire (14), which evaluates mobility, self-care ability, daily activities, presence of pain, anxiety and depression, and includes a self-rated health score ranging from 0 to 100. Both scales are available in the Supplementary Material (**Supplementary Tables S1, S2**).

### 2.3 Statistics

Due to the epidemiological and cross-sectional design of the study, variables were primarily analyzed using descriptive statistics. Analyses were conducted using R statistical software (version 4.2.1, R Development Core Team, 2022). Quantitative data were reported as mean ± standard deviation (SD), while qualitative data were presented as absolute counts and percentages, excluding missing values. Subanalyses of key variables used the χ² or Fisher’s exact test for categorical data and the Student’s t-test for continuous data, following verification of statistical assumptions. A two-tailed p-value <0.05 was considered statistically significant.

## 3 Results

### 3.1 Demographics and diagnostic distribution

A total of 223 participants were included in the study, with a higher proportion of women (61.4%, n=137) than men (38.6%, n=86). The mean age at inclusion was 47.1 years (SD: 18.6). Of the participants, 65.0% (n=145) had PID, while the remaining 35.0% (n=78) were diagnosed with SID. Among those with PID, the most common diagnosis was common variable immunodeficiency (CVID) (39.8%, n=88), followed by IgG subclass deficiency (6.3%, n=14), combined IgA and IgG subclass deficiency (5.9%, n=13), and syndromic immunodeficiencies (5.4%, n=12). Among the participants with SID, the most frequent causes included immunosuppressive therapy and chemotherapy due to lymphoma (33.3%, n=26), myeloma (24.4%, n=19), autoimmune diseases (12.8%, n=10), and leukemia (6.4%, n=5).

### 3.2 Autoimmune manifestations, organ involvement and neoplasia

Patients with PID included 54 men (42.5%) and 73 women (57.5%). Their mean age was 41.05 (SD 17.52) years old. Autoimmune cytopenia was present in 14.2% of participants. Regarding organ-specific autoimmune disease, 12 patients (9.45%) had hypothyroidism and 11 patients (7.8%) had arthritis. There were no patients with type 1 diabetes mellitus. Among systemic autoimmune diseases, 2.4% had Sjögren syndrome. Notably, lymph node or spleen affectation was documented in 15.75% (n=20). Liver disease was present in 21 patients (16.54%). Bronchiectasis and non-infectious lung involvement was present in 42 (33.07%) and 31 (24.41%) patients, respectively. Enteropathy was noted in 25.2% (n=32). Skin disease was present in 24.41% (n=31).

Among patients with SID, 27 were men (34.6%) and 51 were women (65.4%). They were significantly older than those with PID, with a mean age of 58.72 (SD 15.26) years old (p<0.0001). Hematologic neoplasias were present in 61.90% (n=39) and solid tumor was documented in 11.11% patients (n=7). Four patients (6.35%) had received a solid organ transplantation and other 15 patients (23.81%) had received HSCT.

### 3.3 Treatment with SCIg

One hundred and fourteen patients (52.05%) of the cohort were receiving SCIg 20%, and 105 (47.95%) were receiving fSCIg 10%. When examining the Ig preparations, Hyqvia^®^ (n=105, 47.95%) was the most commonly used (n=102, 46.57%), followed by Hizentra^®^ (n=102, 46.57%) and Xembify^®^ (n=12, 5.48%). There were no patients receiving push-SCIg therapy. Previous treatment with IVIg was documented in 148 (66.67%) patients.

Serum IgG levels prior treatment initiation were 563.01 mg/dL (SD 348.14), while the mean IgG trough levels during treatment was 1,041.16 mg/dL (SD 361.63). The average SCIg total week dose administered was 8.04 g (SD: 2.87). The dosing frequency for SCIg varied, with 40.19% (n=86) receiving weekly infusions, 11.21% every two weeks (n=25), 28.97% (n=62) every three weeks, and 18.69% (n=40) monthly. Patients typically used an average of 1.86 injection points (SD: 0.38) per infusion. The most common injection site for SCIg was the abdomen, used by 61.26% (n=136) of participants, followed by alternating between the abdomen and thighs (22.07%, n=49). Injection exclusively in the thighs was reported by 13.51% (n=30), while 1.35% (n=3) alternated injection sites between the arms, abdomen, and thighs.

**Figure 2:**
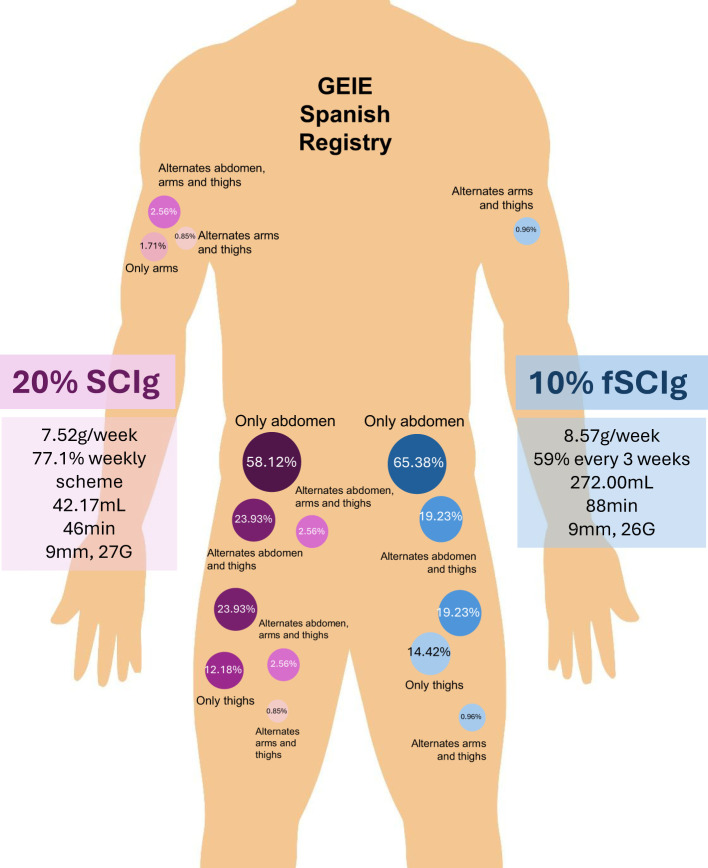
Injection site distribution and administration characteristics of subcutaneous immunoglobulin (SCIg) therapy in GEIE Spanish Registry.

Two patients alternated arms and thighs (0.90%) and another two patients used only the arms (0.90%).The mean abdominal perimeter in our cohort was 89.49cm (SD: 14.85). Regarding needle length, the majority (78.83%, n=175) used 9 mm needles, followed by 12 mm needles (9.01%, n=20), 8 mm needles (6.31%, n=14), 10 mm needles (3.15%, n=7) and 6 mm needles (2.70%, n=6). The majority of the needles used were 26 G (58.37%, n=129) and 27 G (41.63%, n=92). A summary of the results is represented in **Figure 2**.

Patients attended an average of 3 educational sessions (SD: 1.14) for training on SCIg self-administration. Only one patient of the whole cohort administered SCIg in the hospital, while the rest, administered them at home. The average distance from the patient’s residence to the hospital was 31.14 km (SD: 40.13).

When comparing 20% SCIg versus 10% fSCIg characteristics (**Table 1**), age did not differ significantly between groups nor did the sex distribution. The average weekly SCIg dose was significantly lower in the 20% SCIg group (7.52 g/week, SD: 2.21) compared to the 10% fSCIg group (8.57 g/week, SD: 3.34; p = 0.007). Although these differences were uniformly distributed across PID and SID, the mean weekly dose did not significantly differ between PID and SID for either formulation (**Table 2**). No significant differences were found when comparing IgG trough levels for both types of SCIg. Mean infusion volume for 20% SCIg was 42.17 mL (SD: 15.54) and 272mL (SD: 83.65) for 10% fSCIg (p < 0.0001). Conversely, average infusion time was significantly longer for 10% fSCIg (88.31 minutes, SD: 56.85) than for 20% SCIg (46.08 minutes, SD 17.07; p < 0.0001). Most patients receiving SCIg 20% (77.1%) were on a weekly dosing schedule, with 22.9% receiving infusions biweekly. In contrast, the majority of 10% fSCIg recipients followed less frequent regimens, with 59.0% on a three-week cycle and 38.1% on a monthly schedule. However, isolated cases of 10% fSCIg users required weekly or biweekly administration. No significant difference was observed in the number of injection points per infusion.

**Table 1:** Demographic, clinical, and administration characteristics of patients treated with 20% vs. 10% facilitated subcutaneous immunoglobulin (fSCIg).

		SCIg 20% N (%), mean (SD)	fSCIg 10% N (%), mean (SD)	p-value
Age (years)		48.52 (SD 19.72)	45.57 (SD 17.53)	0.24
Female sex		64 (64.65%)	51 (56.04%)	0.29
Immune deficiency				
	PID	77 (53.1%)	68 (46.9%)	1
	SID	41 (52.6%)	37 (47.4%)	
Average SCIg total week dose (g/weeks)		7.52 (SD 2.21)	8.57 (SD 3.34)	0.007
IgG trough levels		1056.96 (SD 415.89)	1023.79 (SD 291.66)	0.508
Mean infusion volume (mL)		42.17 (SD 15.54)	272.00 (SD 83.65)	<0.0001
Average infusion time (minutes)		46.08 (SD 17.07)	88.31 (SD 56.85)	<0.0001
Injection points per infusion		1.83 (SD 0.44)	1.90 (SD 0.29)	0.139
Posology				
	Every week	84 (77.1%)	2 (1.9%)	<0.0001
	Every two weeks	24 (22.85)	1 (1%)	
	Every three weeks	0	62 (59%)	
	Monthly	0	40 (38.1%)	
Injection site				
	Abdomen	68 (58.12%)	68 (65.38%)	0.35
	Thighs	15 (12.82%)	15 (14.42%)	
	Arms	2 (1.71)	0	
	Alternation abdomen and thighs	28 (23.93%)	20 (19.23%)	
	Alternation arms and thighs	1 (0.85%)	1 (0.96%)	
	Alternation abdomen, arms and thighs	3 (2.56%)	0	
Needle length (mm)				
	6 mm	2 (1.71%)	4 (3.85%)	0.0002
	8 mm	14 (11.97%)	0	
	9 mm	83 (70.94%)	91 (87.50%)	
	10 mm	7 (5.98%)	0	
	12 mm	11 (9.40%)	9 (8.65%)	
Needle thickness (G)				
	26 G	31 (26.50%)	98 (94.23%)	<0.0001
	27 G	86 (73.50%)	6 (5.77%)	
Abdominal perimeter (cm)		89.61 (SD 15.39)	89.37 (SD 14.37)	0.91
Educational lessons (number)		2.72 (SD 1.00)	3.32 (SD 1.22)	<0.0001
Average distance to hospital (km)		33.80 (SD 38.39)	28.12 (SD 42.00)	0.30

G, gauge; PID, primary immunodeficiency; deviation; SID, secondary immunodeficiency.

**Table 2:** Comparison of demographic, clinical, and treatment characteristics between primary immunodeficiency (PID) and secondary immunodeficiency (SID) patients across different subcutaneous immunoglobulin (SCIg) formulations.

	PID N (%), mean (SD)	SID N (%), mean (SD)	P-value
Age	41.05 (SD 17.52)	58.72 (SD 15.26)	<0.0001
Female sex	73 (57.7%)	51 (65.4%)	0.391
IgG trough levels (mg/dL)	1044.09 (SD 344.40)	1035.30 (SD 396.39)	0.869
20% SCIg	77 (53.1%)	41 (52.6%)	1
10% fSCIg	68 (46.9%)	37 (47.4%)	
SCIg dose (grams)			
20% SCIg	7.55 (SD 2.30)	7.47 (SD 2.05)	0.708
10% fSCIg	8.50 (SD 3.31)	8.72 (SD 3.46)	
Subjective health perception (0-100)	74.81 (SD 14.80)	68.57 (SD 17.74)	0.008

Regarding injection sites, similar proportions of patients used the abdomen as the primary site. Needle characteristics varied, with 20% SCIg patients predominantly using 9 mm length (70.94%), while a greater variety of needle lengths were observed in the 10% fSCIg group. Needle thickness differed notably, with 26G needles predominantly used in 10% fSCIg (94.23%), whereas SCIg 20% patients primarily used 27G (73.50%; p < 0.0001). There was no significant difference in abdominal perimeter or distance to hospital. However, 10% fSCIg patients required a significantly higher number of educational lessons (3.32, SD 1.22) compared to those receiving 20% SCIg (2.72, SD 1.00; p < 0.0001).

### 3.4 Adverse events

Adverse reactions, both local and systemic, were assessed in patients receiving 20% versus 10% fSCIg, with no overall differences observed (p=0.16 and p=0.08, respectively). However, detailed analysis revealed that certain local adverse reactions, such as local rash (58.65% vs. 36.94%, p=0.002) and pruritus (59.62% vs. 38.74%, p=0.004), were significantly more frequent in the 10% fSCIg group. Systemic reactions, including headache (25.96% vs. 13.68%, p=0.03) and fever (10.58% vs. 1.71%, p=0.01), were also notably higher in patients receiving 10% fSCIg. Additional details on adverse reactions are presented in **Table 3**.

**Table 3:** Local and systemic adverse events in patients treated with 20% vs. 10% fSCIg.

	Grupo 20% SCIg N (%), mean (SD)	Grupo 10% fSCIg N (%), mean (SD)	p-value
Local adverse events	76 (68.47%)	81 (77.88%)	0.16
Local rash	41 (36.94%)	61 (58.65%)	0.002
Pain	23 (20.72%)	28 (26.92%)	0.36
Pruritus	43 (38.74%)	62 (59.62%)	0.004
Swelling	50 (45.05%)	61 (58.65%)	0.06
Nodule	8 (7.21%)	4 (3.85%)	0.44
Leakage	7 (6.31%)	11 (10.58%)	0.37
Hematoma	11 (9.91%)	7 (6.73%)	0.55
Ulcer	3 (2.70%)	1 (0.96%)	0.66
Necrosis	4 (3.60%)	4 (3.85%)	1.00
Systemic adverse events	29 (24.79%)	38 (36.54%)	0.08
Headache	16 (13.68%)	27 (25.96%)	0.03
Asthenia	13 (11.11%)	16 (15.38%)	0.46
Fever	2 (1.71%)	11 (10.58%)	0.01
Arthromyalgia	13 (11.11%)	10 (9.62%)	0.89
Systemic Rash	4 (3.42%)	2 (1.92%)	0.79

Local adverse events were investigated concerning the simultaneous use of antiplatelet agents or oral and subcutaneous anticoagulants. No significant differences were observed in the incidence of local adverse effects between patients receiving SCIg who were also undergoing antiplatelet or anticoagulant therapy and those who were not. The occurrence of local reactions, including swelling, rash, and pain, was comparable across all groups. Further details are represented in **Tables 4**, **5**.

**Table 4:** Local adverse events in relation to antiplatelet agent use.

	Antiplatelet agent use N (%), mean (SD)	No antiplatelet agent use N (%), mean (SD)	p-value
Local adverse events	7 (53.85%)	144 (73.47%)	0.23
Local rash	4 (30.77%)	92 (46.94%)	0.40
Pain	3 (23.08%)	46 (23.47%)	1.00
Pruritus	4 (30.77%)	95 (48.47%)	0.34
Swelling	6 (46.15%)	101 (51.53%)	0.93
Nodule	0 (0.00%)	10 (5.10%)	0.87
Leakage	1 (7.69%)	16 (8.16%)	1.00
Hematoma	1 (7.69%)	17 (8.67%)	1.00
Ulcer	1 (7.69%)	3 (1.53%)	0.60
Necrosis	0 (0.00%)	8 (4.08%)	1.00

**Table 5:** Local adverse events in relation to anticoagulant use.

	Anticoagulant use N (%), mean (SD)	No anticoagulant use N (%), mean (SD)	p-value
Local	11 (68.75%)	145 (73.23%)	0.92
Local rash	5 (31.25%)	96 (48.48%)	0.29
Pain	4 (25.00%)	46 (23.23%)	1.00
Pruritus	8 (50.00%)	96 (48.48%)	1.00
Swelling	6 (37.50%)	105 (53.03%)	0.35
Nodule	0 (0.00%)	12 (6.06%)	0.65
Leakage	0 (0.00%)	18 (9.09%)	0.43
Hematoma	1 (6.25%)	17 (8.59%)	1.00
Ulcer	0 (0.00%)	4 (2.02%)	1.00
Necrosis	0 (0.00%)	8 (4.04%)	0.89

### 3.5 Socieconomic status and health perception

In terms of employment, 40% (n=88) were employed, 14.55% (n=32) were students, and 16.82% (n=37) had a disability allowance. The remaining participants were either retired (24.09%, n=53) or unemployed (4.55%, n=10).

The Gijón Scale (13) was applied to assess family situation, monthly income, housing conditions, social relationships, and social network support (**Table 6**). Nearly half (47.06%) of patients live independently with family, and 31.67% reside with a spouse of similar age. Economically, most patients (51.64%) report incomes above 1.5 times the minimum wage, while 15.49% earn below the minimum contributory pension. All patients have adequate housing, and a substantial majority (94.42%) maintain active social relationships, with 99.54% reporting support from family or neighbors.

**Table 6:** Gijón scale categories: family situation, economic situation, housing situation, social relationships, and social support network.

Category	Description	Frequency (n, %)
Family Situation	Lives with family without physical/psychological dependence	104 (47.06%)
	Lives with spouse of similar age	70 (31.67%)
	Lives with family and/or spouse with some dependency	23 (10.41%)
	Lives alone, has nearby children	10 (4.52%)
	Lives alone, no children or children live far away	14 (6.33%)
Economic Situation	Income more than 1.5 times the minimum wage	110 (51.64%)
	Income from 1.5 times the minimum wage to the minimum wage inclusive	44 (20.66%)
	Income from minimum wage to minimum contributory pension	14 (6.57%)
	Non-contributory pension	12 (5.63%)
	No income or income below the aforementioned threshold	33 (15.49%)
Housing Situation	Adequate to needs	0 (0%)
	Architectural barriers at home or building entrance (e.g., stairs, narrow doors, bathrooms)	0 (0%)
	Dampness, poor hygiene, inadequate equipment (e.g., no complete bathroom, no hot water, no heating)	0 (0%)
	No elevator, no telephone	0 (0%)
	Inadequate housing (e.g., shanty, home declared in ruins, absence of basic amenities)	0 (0%)
Social Relationships	Active social relationships	203 (94.42%)
	Social relationships only with family and neighbors	7 (3.26%)
	Social relationships limited to family or neighbors	5 (2.33%)
	Does not leave home, receives family	0 (0%)
	Does not leave home, no visits received	0 (0%)
Social Support Network	Family and neighborhood support	216 (99.54%)
	Social volunteer support or home assistance	1 (0.46%)
	No support	0 (0%)
	Awaiting admission to geriatric residence	0 (0%)
	Permanent care required	0 (0%)

Additionally, EuroQol-5D-3L questionnaire (14) was also applied for the evaluation of mobility, self-care ability, daily activities, presence of pain, anxiety and depression. Patients generally experience few limitations. Most report no issues with mobility (79.72%) and personal care (89.86%), with a large proportion (77.42%) able to perform daily activities independently. Pain is minimal for 66.82%, and anxiety affects only 21.40% at moderate levels, with severe cases being rare (1.40%). The average subjective health rating stands at 72.69/100. Significant differences were found in this subjective health perception between PID and SID, with SID patients having significantly lower scores (68.57/100) than PID patients (74.81/100) (p=0.008) (**Table 2**). Further details are represented in **Table 7**.

**Table 7:** EuroQol-5D-3L scale dimensions: mobility, personal care, daily activities, pain/discomfort, anxiety/depression, and subjective health perception.

Dimension	Response	Frequency - N (%)
Mobility	No problems walking	173 (79.72%)
	Some problems walking	38 (17.51%)
	Confined to bed	6 (2.76%)
Personal Care	No problems with personal care	195 (89.86%)
	Some problems washing or dressing	14 (6.45%)
	Unable to wash or dress	8 (3.69%)
Daily Activities	No problems with daily activities (e.g., work, study, household tasks)	168 (77.42%)
	Some problems with daily activities	40 (18.43%)
	Unable to perform daily activities	9 (4.15%)
Pain/Discomfort	No pain or discomfort	141 (66.82%)
	Moderate pain or discomfort	68 (32.23%)
	Unable to perform daily activities	2 (0.95%)
Anxiety/Depression	Not anxious or depressed	166 (77.21%)
	Moderately anxious or depressed	46 (21.40%)
	Severely anxious or depressed	3 (1.40%)
Subjective health feeling (0-100)		72.69 (SD 16.09)

## 4 Discussion

This study provides important real-world data on the use of different SCIg formulations in patients with PID and SID across multiple centers in Spain. Our findings confirm the overall effectiveness of SCIg in maintaining adequate IgG levels and demonstrate a favorable safety profile, with no significant differences in overall adverse event rates between the 10% and 20% formulations. Ten percent SCIg was linked to higher frequencies of certain mild local and systemic adverse events, such as local rash and fever, yet its dosing schedule resulted in less disruption to daily life. Quality-of-life assessments revealed minimal limitations in daily activities, underscoring SCIg potential to enhance patient autonomy and satisfaction. These results highlight the need for individualized treatment strategies and underscore the vital role of expert immunodeficiency nursing teams in ensuring safe and effective SCIg administration.

Most real-world studies on SCIg focus on small, highly homogeneous subgroups of patients, either with PID or SID, or those receiving a specific type of Ig, either 10% or 20% formulations (15–22). This approach does not accurately reflect everyday practice in specialized nursing units or outpatient immunodeficiency clinics, where both types of immunodeficiencies and different Ig formulations are commonly managed. By including all four patient subgroups, our study extends the applicability of SCIg recommendations to both younger and older patients, as demonstrated by the significantly higher mean age for SID in our cohort compared to PID, and to patients with diverse and complex comorbidities. These include immune dysregulation inherent to certain PIDs, patients who have undergone chemotherapy regimens, those who have received hematologic or solid organ transplants, and individuals on anticoagulant or antiplatelet therapy. Our findings provide consistent evidence on the tolerability, efficacy, and quality-of-life impact of SCIg across different formulations for a broader patient population.

Comparing these findings to prior studies, this study reinforces previous evidence that SCIg therapy, independently of its formulation, is an effective alternative to IVIg for achieving adequate IgG levels in patients with immunodeficiency (5, 6). Our findings align with previous real-world data, showing that 10% fSCIg is typically administered every three weeks or monthly, resulting in a lower treatment burden and minimal interference with daily activities, while 20% SCIg is administered on a weekly to biweekly schedule (18, 21). No significant differences in IgG trough levels were observed between the 20% and 10% SCIg groups, despite the significantly lower average weekly dose in the 20% SCIg group, regardless of PID or SID status. This outcome may be explained by pharmacokinetic differences between the formulations and suggests potential cost-effectiveness in specific contexts, even though more frequent monthly injections and infusion sessions are required.

Most patients also used a mean of 2 sites per infusion in our cohort (21). However, needle length and caliber was relatively lower in our report than in previous experiences for both presentations (15, 18–20). Patients in our cohort treated with 10% fSCIg formulations required more educational sessions for self-administration, consistent with previous findings (16). Consequently, 20% SCIg may be a more suitable and simpler option for patients who have relatively more difficulty with self-administration. These results underscore the importance of a dedicated nursing team in educating and supporting patients using SCIg.

In recent years, the transition from IVIg to SCIg has been promoted, as SCIg administration offers significant advantages, including reduced systemic adverse reactions and the convenience of home-based treatment, both of which have been shown to improve patient satisfaction and autonomy (23–25). Despite its benefits, the use of SCIg in Spain remains suboptimal. This is largely due to the long-standing preference for IVIg, limited training among healthcare professionals in SCIG administration, established IVIg-specific infrastructure, and a general lack of familiarity with SCIG protocols. Data from the Spanish GTEM-SEMI-CVID registry reveal that the majority of CVID patients in Spain continue to receive IVIg, underscoring the need for sustained efforts to facilitate the transition to SCIg (9). In our cohort, 66% of patients were previously treated with IVIg and have continued this regimen, while the remaining patients initiated SCIg directly following the identification of the need for IgRT.

It is essential to recognize that SCIg administration can be associated with both local and systemic adverse effects. The variation in adverse reactions observed between different SCIg formulations in our study aligns with international findings, suggesting that formulation concentration may significantly influence patient tolerance and comfort (6). Our study reports one of the highest rates of local adverse events compared to other cohorts (12, 18, 22, 26), likely due to the inclusion of typically expected local infusion reactions associated with subcutaneous administration. Moreover, we observed a notably high rate of systemic adverse events, potentially due to the large proportion of patients using 10% fSCIg in our cohort, compared to other studies where the sample size for 10% fSCIg users was limited (12). Additionally, this information was collected through patient-reported questionnaires as part of our patient-experience-centered approach, capturing cumulative experiences over time rather than measured data from a specific observational period. Consequently, direct comparisons with other studies may be limited.

We have reported that facilitated 10% fSCIg has been related to the development of more local adverse events than 20% SCIg, as previously described (15, 16); in our case, specifically, pruritus and rash. When exploring the relationship between the development of local complications and the concomitant use of antiplatelet or anticoagulant therapies, no significant differences were found. This concurs with previous evidence and supports the safety of SCIg in these groups of patients (27, 28). Additionally, 10% fSCIg was significantly associated with the development of systemic adverse events when compared to 20% SCIg in our cohort. In this regard, we have noted that fever and headache have been significantly more frequently observed in the 10% fSCIg group, as documented by other studies (17, 18, 29). In fact, in the FIGARO study with 156 patients, systemic adverse reactions were reported in 7.4% of all visits, with flu-like symptoms, fatigue, fever, and headache being the most commonly reported reactions (18). However, some reports have not documented this difference, possible related to very low sample size (16). Interestingly, in a study including 327 patients with CVID receiving IGRT under several administration routes reported similar degrees of concern regarding IgRT related adverse events independently of the administration route (30). This highlights the need for thoroughly informing patients about these differences in adverse reactions so they can collaboratively select the preferred route of administration, ensuring their choice aligns with their tolerance and comfort preferences.

The analysis of socioeconomic status and health perception offers valuable insights into the well-being and characteristics of the patient population receiving SCIg, as previously reported in studies devoted to the quality of life in patients undergoing this therapy (23–25). Employment data show a diverse mix of active workers, students, individuals on disability allowances, and retirees, reflecting varying levels of economic independence within this cohort. Despite these differences, the Gijón Scale demonstrates overall socioeconomic stability, with the majority of patients living in adequate housing, maintaining active social connections, and benefiting from strong family and neighborhood support. Additionally, caregiver involvement is minimal due to the high degree of patient autonomy achieved through home-based SCIg administration, which is facilitated by comprehensive educational programs provided by expert nursing teams.

Health perception, evaluated using the EuroQol-5D-3L, shows generally positive outcomes, with few limitations in mobility, personal care, or daily activities, and a low incidence of severe pain or discomfort, irrespective of socioeconomic status. This stability is likely supported by Spain’s comprehensive public healthcare system, which ensures free access to nursing and medical care, as well as to all necessary consumables, SCIg, and infusion pumps. These findings underscore the economic, social, and educational characteristics of this patient population and the crucial role of structured support systems in promoting long-term adherence and well-being.

While anxiety and depression are present, they are generally moderate. These findings suggest that patients demonstrate resilience in daily functioning, though ongoing psychological and social support remains essential to address the needs of those experiencing significant discomfort or mental health challenges. The average subjective health rating of 72.69 out of 100 further reflects a positive but improvable perception of well-being. However, significant differences in subjective health perception were observed between PID and SID patients, with SID patients reporting lower scores. This underscores the need to address not only social and economic factors but also the impact of comorbidities and the type of immunodeficiency to enhance patient adherence and satisfaction with SCIg. Multidisciplinary support—particularly from expert nursing teams—plays a critical role in tailoring care and optimizing long-term outcomes for this diverse patient population.

The study has several strengths. Its multicentric approach, involving diverse immunodeficiency units across Spain, enhances the generalizability of findings and captures a comprehensive patient population with varied backgrounds and health status. The large sample size and standardized data collection through the GEIE Spanish Registry provide robust evidence of SCIg’s real-world effectiveness and tolerance in patients with PID and SID. Additionally, the focus on both clinical outcomes and socio-economic impact is unique, offering a holistic view of SCIg’s benefits and challenges in this population.

However, this study has limitations that should be noted. The retrospective design may introduce biases, as certain patient details and adverse reactions may have been overreported or inconsistently documented, and also affected by memory bias. Additionally, the absence of a control group treated with IVIg limits the ability to directly compare the efficacy and safety of SCIg versus IVIg within this cohort. In this regard, the study did not investigate the reasons for transitioning from IVIg to SCIg, which remains an important area for future exploration. Moreover, a prospective record of the number of mild and severe or recurrent infections was not performed, and we only assessed effectiveness in maintaining IgG trough levels. The absence of a pediatric population also limits the evidence of this study to patients over 14 years old. Lastly, although the multicentric design strengthens generalizability, variations in training and administration practices across centers could influence patient outcomes and perceptions of SCIg therapy.

## 5 Conclusion

This multicenter study provides valuable real-world insights into the use of SCIg therapy in patients with PID and SID across Spain. Our findings confirm SCIg effectiveness in maintaining adequate IgG levels and its adequate safety profile, along with the associated benefits of increased patient autonomy and quality of life. The study emphasizes the need for tailored treatment strategies that consider not only the formulation and potential adverse effects but also factors such as dosing schedules, patient preferences, and the specific clinical and socioeconomic profile of each individual. The socioeconomic assessment highlights that most patients benefit from stable housing and robust social support networks, which, together with Spain’s comprehensive public health system, may contribute to the generally positive health perceptions observed. Understanding the factors influencing the transition from IVIg to SCIg, including social, demographic, and patient preferences, represents a valuable area of research for future studies and for establishing switching protocols. Overall, our results underscore the importance of personalized care and the role of dedicated nursing teams in supporting optimal outcomes and patient satisfaction with SCIg therapy.

## Data Availability

The raw data supporting the conclusions of this article will be made available by the authors, without undue reservation.
