# Supplementary material for: Patient-centered outcomes with subcutaneous immunoglobulin use for infection control in primary and secondary immunodeficiencies: data of a GEIE Spanish Registry
Source: Front Immunol. 2025 Feb 14;16:1532367. doi: 10.3389/fimmu.2025.1532367 (PMC11868073; doi:10.3389/fimmu.2025.1532367)
Supplement: Supplementary file 1 [file DataSheet1.docx]

**Supplementary Material**

**Table S1.** Gijón scale

| Category | Description | Punctuation |
| --- | --- | --- |
| Family Situation | Lives with family without physical/psychological dependence | 1 |
|  | Lives with spouse of similar age | 2 |
|  | Lives with family and/or spouse with some dependency | 3 |
|  | Lives alone, has nearby children | 4 |
|  | Lives alone, no children or children live far away | 5 |
| Economic Situation | Income more than 1.5 times the minimum wage | 1 |
|  | Income from 1.5 times the minimum wage to the minimum wage inclusive | 2 |
|  | Income from minimum wage to minimum contributory pension | 3 |
|  | Non-contributory pension | 4 |
|  | No income or income below the aforementioned threshold | 5 |
| Housing Situation | Adequate to needs | 1 |
|  | Architectural barriers at home or building entrance (e.g., stairs, narrow doors, bathrooms) | 2 |
|  | Dampness, poor hygiene, inadequate equipment (e.g., no complete bathroom, no hot water, no heating) | 3 |
|  | No elevator, no telephone | 4 |
|  | Inadequate housing (e.g., shanty, home declared in ruins, absence of basic amenities) | 5 |
| Social Relationships | Active social relationships | 1 |
|  | Social relationships only with family and neighbors | 2 |
|  | Social relationships limited to family or neighbors | 3 |
|  | Does not leave home, receives family | 4 |
|  | Does not leave home, no visits received | 5 |
| Social Support Network | Family and neighborhood support | 1 |
|  | Social volunteer support or home assistance | 2 |
|  | No support | 3 |
|  | Awaiting admission to geriatric residence | 4 |
|  | Permanent care required | 5 |

Table S2. EuroQol-5D-3L scale

| Dimension | Response (mark with X) |  |
| --- | --- | --- |
| Mobility | No problems walking |  |
|  | Some problems walking |  |
|  | Confined to bed |  |
| Personal Care | No problems with personal care |  |
|  | Some problems washing or dressing |  |
|  | Unable to wash or dress |  |
| Daily Activities | No problems with daily activities (e.g., work, study, household tasks) |  |
|  | Some problems with daily activities |  |
|  | Unable to perform daily activities |  |
| Pain / Discomfort | No pain or discomfort |  |
|  | Moderate pain or discomfort |  |
|  | Unable to perform daily activities |  |
| Anxiety / Depression | Not anxious or depressed |  |
|  | Moderately anxious or depressed |  |
|  | Severely anxious or depressed |  |
| Subjective health feeling (0-100) |  | |
